# Knockdown of pp32 Increases Histone Acetylation and Ameliorates Cognitive Deficits

**DOI:** 10.3389/fnagi.2017.00104

**Published:** 2017-04-20

**Authors:** Qiong Feng, Gao-Shang Chai, Zhi-Hao Wang, Yu Hu, Dong-Sheng Sun, Xiao-Guang Li, Rong-Hong Ma, Yi-Rong Li, Dan Ke, Jian-Zhi Wang, Gong-Ping Liu

**Affiliations:** ^1^Key Laboratory of Ministry of Education of China for Neurological Disorders, Department of Pathophysiology, Collaborative Innovation Center for Brain Science, School of Basic Medicine, Tongji Medical College, Huazhong University of Science and TechnologyWuhan, China; ^2^Department of Basic Medicine, Wuxi Medical School, Jiangnan UniversityWuxi, China; ^3^Department of Laboratory Medicine, Tongji Medical College, Union Hospital, Huazhong University of Science and TechnologyWuhan, China; ^4^Department of Laboratory Medicine, Zhongnan Hospital, Wuhan UniversityWuhan, China; ^5^Co-Innovation Center of Neuroregeneration, Nantong UniversityNantong, China

**Keywords:** pp32, cognition, histone acetylation, aging, dendritic complexity

## Abstract

Aging is a cause of cognitive decline in the elderly and the major risk factor for Alzheimer's disease, however, aging people are not all destined to develop into cognitive deficits, the molecular mechanisms underlying this difference in cognition of aging people are obscure. Epigenetic modifications, particularly histone acetylation in the nervous system, play a critical role in regulation of gene expression for learning and memory. An inhibitor of acetyltransferases (INHAT) is reported to suppress histone acetylation via a histone-masking mechanism, and pp32 is a key component of INHAT complex. In the present study, we divided ~18 m-old aged mice into the cognitive-normal and the cognitive-impaired group by Morris water maze, and found that pp32 level was significantly increased in the hippocampus of cognitive-impaired aged mice. The mRNA and protein levels of synaptic-associated proteins decreased with reduced dendrite complexity and histone acetylation. Knockdown of pp32 rescued cognitive decline in cognitive-impaired aged mice with restoration of synaptic-associated proteins, the increase of spine density and elevation of histone acetylation. Our study reveals a novel mechanism underlying the aging-associated cognitive disturbance, indicating that suppression of pp32 might represent a promising therapeutic approach for learning and memory impairments.

## Introduction

In the twenty-first century, aging population has emerged as a major demographic trend worldwide. Declining fertility, improved health, and longevity, have swelled the older populations dramatically. The global population of people aged 60 and over representing 11 percent of the world's population, were 680 million people in 2009. They have increased by 10.4 million just since 2007—an average increase of 30,000 each day. By 2050, the 60 and older population will increase from 11 to 22 percent of the world's population-increasing from 680 million to 2 billion. Aging is the major risk factor for major neurodegenerative diseases, such as Alzheimer's disease, Parkinson's disease, and amyotrophic lateral sclerosis. Most people over the age of 70 exhibit some degree of cognitive decline, particularly for short-term memory, and this accelerates with aging. However, some old people show no impaired learning and memory abilities. The molecular mechanisms underlying this difference in cognition of aged people are obscure.

Using histone acetylation to remodel chromatin is a key mechanism to control gene expression (Jenuwein and Allis, 2001). The electrostatic affinity between neighboring histones and DNA is diminished by histone acetylation, and consequently, histone acetylation can promote a more open chromatin structure that allows for memory-related genes transcription (Brownell and Allis, 1996). Epigenetic modifications, particularly histone acetylation in the central nervous system, play a critical role in regulation of learning and memory-related genes expression (Kosik et al., 2012). A number of studies indicate that aging correlates with brain region-specific changes of gene expression (Lee et al., 1999; Lu et al., 2004; Xu et al., 2007; Berchtold et al., 2008), and altered histone acetylation contributed to aging-associated changes in gene expression and cognitive decline (Peleg et al., 2010). Therefore, we hypothesized that altered histone acetylation might also contribute to the cognitive differences in aging people.

Acetylation and deacetylation of histone is catalyzed by histone acetyltransferases (HATs) and histone deacetylases (HDACs), respectively. Recently, independently of changes of HATs or HDACs, a cellular complex termed inhibitor of acetyltransferases (INHAT) was reported to regulate histone acetylation (Seo et al., 2002). INHAT suppresses histone acetylation through a mechanism namely histone-masking, in which INHAT binds to histones and masks their access to acetyltransferases (Seo et al., 2001). As an endogenous inhibitor of protein phosphatase 2A (PP2A) (Li et al., 1996), ANP32A also named ${\text{I}}_{1}^{\text{PP}2\text{A}}$, pp32, and so on, is one of the major subunits of INHAT (Seo et al., 2002). Of note, the reduction of PP2A activity leading to hyperphosphorylation of a microtubule associated protein tau plays an important role in neurodegeneration in Alzheimer's disease (AD) (Chen et al., 2008; Tanimukai et al., 2009). Interestingly, pp32 level is selectively up-regulated in the areas of AD brain, which affected with neurofibrillary pathology (Tanimukai et al., 2005). Therefore, regarded as an inhibitor of PP2A, pp32 has been causally linked to neurodegeneration in AD (Tanimukai et al., 2005).

In the present study, we examined the impact of pp32 on cognitive performance in a mouse model of aging. We demonstrated that pp32 level was inversely correlated with cognitive performance. Knockdown of pp32 increased histone acetylation and promoted the expression of genes associated with learning and memory, which resulted in rescuing of cognitive decline. Our study suggests that pp32 may represent an early biomarker of an impaired genome-environment interaction in aging.

## Materials and methods

### Antibodies

Polyclonal antibody (pAb) anti-H4K8 (acetylated histone H4 at lysine 8), monoclonal antibody (mAb) anti-tau-5 (total tau), mAb anti-tau-1 (unphosphorylated tau at Ser198/199/202), pAb anti-NR2A, pAb anti-synapsin-1, pAb anti-synaptophysin, pAb anti-GluR2, and monoclonal antibody (mAb) anti-GluR1, were purchased from Millipore (Billerica, MA). PAb anti-H4K12 (acetylated histone H4 at lysine 12), mAb anti-β-actin, pAb anti-RFP, pAb anti-NR2B, pAb anti-pp32, pAb anti-Histone H4, pAb anti-Histone H3, and pAb anti-H3K9 (acetylated Histone H3 at lysine 9) were from Abcam (Cambridge, USA). pAb anti-pT205 (phosphorylated tau at Thr205), pAb anti-pS396 (phosphorylated tau at Ser396), and pAb anti-pS404 (phosphorylated tau at Ser404) were from SAB (Pearland, TX). PAb anti-H3K14 (acetylated Histone H3 at lysine 14) was come from Cell Signaling (Cambridge, MA). MAb anti-DM1A (total α-tubulin) was purchased from Sigma (St. Louis, MO).

### Animals

Male C57 mice (18 months old) were purchased from the animal center of Tongji Medical College, Huazhong University of Science and Technology. All mice were kept at 24 ± 2°C on daily 12 h light-dark cycles with *ad libitum* access to food (supplied by Beijing Huafu Kang Biotechnology Co., Ltd, product number: 1032) and water. By the way, gene expression changes in the course of normal brain aging are sexually dimorphic; the female brain showed fewer gene changes than males overall in aging (Xu et al., 2007; Marintcheva et al., 2008). To avoid the gender related differential expression of the transcriptomes induced by gender, only male mice were used. All animal experiments of this study were performed according to the “Policies on the Use of Animals and Humans in Neuroscience Research” revised and approved by the Society for Neuroscience in 1995, and the Guidelines for the Care and Use of Laboratory Animals of the Ministry of Science and Technology of the People's Republic of China, and the Institutional Animal Care and Use Committee at Tongji Medical College, Huazhong University of Science and Technology approved the study protocol. We made all efforts to minimize animal suffering and sacrifice in this experiment process.

### Behavioral tests

**Figure 1:**
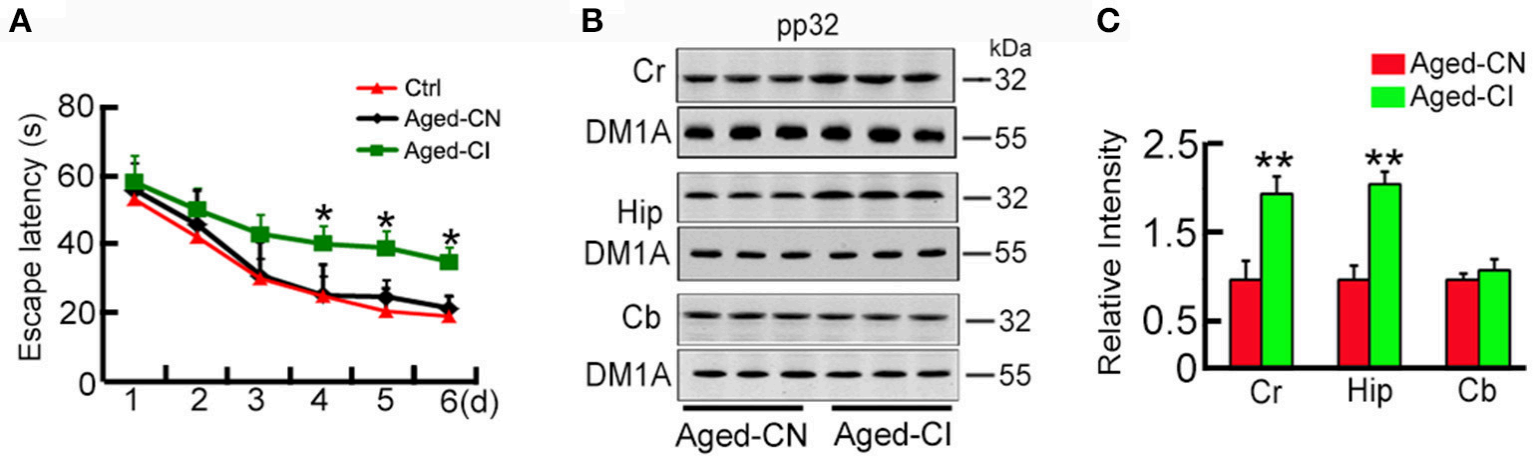
**Pp32 level increases in the hippocampus of aged cognitive-impaired mice. (A)** Escape latency in the Morris water maze task of 12 m-old (Ctrl, *n* = 9), cognition-normal 18 m-old C57 mice (Aged-CN, *n* = 9) and cognitive-impaired 18 m-old C57 mice (Aged-CI, *n* = 9).^*^p < 0.05 vs. Ctrl (mean ± SEM). **(B,C)**.Western blotting analysis **(B)** and quantification **(C)** of pp32 levels in the cortex (Cr), hippocampus (Hip) and cerebellum (Cb) of Aged-CN and Aged-CI mouse brain (*n* = 3 for each group). Tubulin was used as a loading control and was detected with DM1A antibody. Note: pp32 levels are significantly increased in the Hip of Aged-CI, compared to that in Aged-CN. ^*^p < 0.05, ^**^p < 0.01 vs. Aged-CN.

Experimental procedure: 18-month-old C57 mice were divided into cognitive normal (Aged-CN) group and cognitive impaired (Aged-CI) group according to the escape latency after learning through Morris water maze (MWM)-tests. Combined with the escape latency in other days, if the escape latency to find the submerged platform in sixth day is within 20 s, which is similar as that of 12-month-old normal mice, the mouse is grouped into cognitive normal (CN) group, while the mice could not find the platform in 30 s were grouped as cognitive impaired (CI) group (Figure 1A). Eleven CN mice were injected with scrambled control lentivirus and used as Aged-CN-siC group, while 22 CI mice were further divided into two groups (11 each), one group was injected with pp32-shRNA (Aged-CI-sipp32) to knockdown pp32 and another group was injected with the scrambled control (Aged-CI-siC).

Four weeks after brain infusion of the virus, MWM-test was used to test the spatial learning and memory in Aged-CN-siC, Aged-CI-sipp32, and Aged-CI-siC group mice (Morris et al., 1982). For spatial learning, mice were trained in water maze to find a hidden platform for 6 consecutive days, 4 trials per day with a 30 s interval from 14:00 to 20:00 p.m. On each trial, the mice started from one of the four quadrants facing the wall of the pool and ended when the animal climbed on the platform. If the mice did not locate the platform within 60 s, they were guided to the platform. The swimming path and the time used to find the platform (latency) were recorded each day during training. The spatial memory was tested 24 h after the last training. The platform was removed and the times passing through the platform quadrant and the first time to cross the platform (latency) were recorded.

Seven days after the MWM-test, the step-down avoidance test was performed to evaluate the ability of learning and memory (Liu et al., 2013). Briefly, the mice were kept in the cage for 3 min to adapt to the environment before the experiment, and then the mice received training by delivering electrical shock (36 v) for 3 min. The short term memory (STM) and long term memory (LTM) were tested respectively in 15 min and 24 h after the training by measuring the step-down latency in 3 min.

### Brain infusion of lentiviral constructs

The pp32-shRNA sequence is CGGATTTATTTAGAGCTGC and the scrambled control sequence is TTCTCCGAACGTGTCACGT, and all of them were cloned into LentiLox 3.7. The RFP sequence was driven by a cytomegalovirus (CMV) promoter and terminated using polyadenylation signal in the 3′ long terminal repeat (LTR). The third generation packaging systems was used for lentiviral production. The standard procedure was used to construct the RFP-labeled lentiviral pp32-shRNA (LV-sipp32) and the scrambled control (LV-siC). By the calcium phosphate method, the recombinant lentivirus was produced by transient transfection of HEK293T cells. According to the cell status, HEK293T cell supernatant was collected 48 h and 72 h after transfection and centrifuged at 4,000 g for 10 min at 4°C to remove cell debris; and then the supernatant was filtered through a 0.45 μm filter and centrifuged at 25,000 rpm for 2 h at 4°C. After centrifugation, the supernatant was discarded and the pellet was resuspended by the virus preservation solution and continued to centrifuge at 10,000 rpm for 5 min, the supernatant was packaged. Lastly, quantitative PCR (qPCR) was used to detect virus titer.

For brain injections, mice were positioned in a stereotaxic instrument, then 2 μl LV-sipp32 or LV-siC were bilaterally injected into CA3 region of hippocampus (AP ± 2.0, ML −1.5, DV −2.0) at a rate of 0.50 μl/min. The needle was left in place for ~3 min before being withdrawn. This operation did not increase the death rate or change the normal activity of the mice compared with the sham operation group. Western blotting and RT-PCR were used to measure the knockdown efficiency at 4 weeks after the virus injection.

### Real time PCR

Total RNA (3 μg) was isolated by Trizol™ (Invitrogen, CA), and then was reversely transcribed. The produced cDNA (1 μl) was used for real time PCR with primer sets: 5′-TATGCTCTTTGGGTCAGTCTCGTT-3′ and 5′-GTCCCTTTATCCTCCGTCTTTCTT-3′ for NR2B, 5′-TCAAGGAAAGCAGAAGGGGAAA-3′ and 5′-TGTGGAATGGAATGATAGGCGA-3′ for NR2A, 5′-CACATGTAGCCGGAGTGATG-3′ and 5′-CACTCAAGAGGATGGGGAAA-3′ for GluR1, 5′-ATTTCGGGTAGGGATGGTTC-3′ and 5′-ACCATCCTTCACTGGCATTC-3′ for GluR2, 5′-CAAACAATACCGAAGGGCACAG-3′ and 5′-AAGAGGGCTAGATAATCAGAAGACAGA-3′ for synaptophysin, 5′-CTTTGCTTGTTTATTTTGCTTC-3′ and 5′-CCAATGTGTTTATCTGTGACTG-3′ for synapsin I, 5′-CACGTTTTCTCGGTAGGCATT-3′ and 5′-AGGGGACCTGGAAGTATTGGC-3′ for pp32, and 5′-AGCCTTCCTTCTTGGGTAT-3′ and 5′-GCTCAGTAACAGTCCGCCTA-3′ for β-actin.

### PP2A activity assay

PP2A activity was measured using a Serine/Threonine Phosphatase Assay Kit (Promega, MA), which could specifically measure the activity of PP2A through specific substrate and alternative buffer systems. The absorbance was read at 600 nm (BioTek Instruments, VT).

### Western blotting

Western blotting was performed as described previously (Duan et al., 2013). The hippocampal CA3 region (where virus injected), cortex (temporal lobes), and cerebellum were rapidly removed and homogenized at 4°C using a Teflon glass homogenizer with homogenate buffer (Tris-HCl 50 mM (pH 7.4), NaCl 150 mM, NaF 10 mM, Na_3_VO_4_1 mM, EDTA 5 mM, benzamidine 2 mM, and phenylmethylsulfonyl fluoride 1 mM). After mixed with sample buffer (3:1, v/v, containing 200 mM Tris-HCl (pH 7.6), 8% SDS, 40% glycerol, 40 mM dithiothreitol), the extract was boiled for 10 min. The proteins were transferred to nitrocellulose membranes after separated by 10% SDS-polyacrylamide gel electrophoresis. The membranes were blocked for 1 h with 5% nonfat milk, which dissolved in TBSTween-20 (containing 50 mM Tris-HCl (pH 7.6), 150 mM NaCl, 0.2% Tween-20), and probed with primary antibody at 4°C overnight. Finally, the blots were incubated with anti-rabbit or anti-mouse IgG conjugated to IRDyeTM (800 CW) at room temperature and visualized using the Odyssey Infrared Imaging System (Licor biosciences, Lincoln, NE, USA).

### Golgi staining and dendritic morphology analysis

Golgi staining was performed as described previously (Woolley and McEwen, 1992). After anesthetized, the mice were transcardially perfused with 4% paraformaldehyde. Individual sections were incubated overnight at room temperature in water solution of 3.5% K_2_Cr_2_O_7_ and 0.4% OsO_4_, sandwiched in two glass slides and incubated in 1% AgNO_3_ at room temperature in dark for 5 h. Then the slide assemblies were dismantled in water and the sections were mounted on gel-coated slides (0.5% porcine gelatin), and dehydrated in a series of graded ethanol rinses. The sections were cleared with Histoclear and cover slipped with cytoseal. Olympus BX60 (Tokyo) was used to take the images.

The segments of dendrites at a distance of 190–210 μm (distal) from the soma were used to determine the spine density. In order to acquire images for spine analysis, a Zeiss Axioimager microscope with a 63 × oil immersion objective was used to take images of the dendritic segments under bright-field illumination. The spine morphology was analyzed according to a previously reported method (Magariños et al., 2011), which does not assess spine density in a 3 dimensional manner but focuses on spines paralleled to the plane of section. Although the method may underestimate the total number of spines, it facilitates a direct comparison of treatment groups when they are analyzed in an identical manner. Linear spine density, which presented as the number of spines per 10 μm of dendrite length, was calculated using Image J software (Spires-Jones et al., 2011). Spines were classified into the following categories on the basis of morphology: (i) thin: spines with a long neck and a visible small head; (ii) mushroom: big spines with a well-defined neck and a very voluminous head. Data from 5 to 7 neurons were averaged per animal and used in further statistical analysis.

### Immunohistochemistry

In brief, the mice were anesthetized with chloral hydrate (1 g/kg) and perfused through aorta with 0.9% NaCl followed by phosphate buffer containing 4% paraformaldehyde. Brains were removed and postfixed in perfusate overnight, and then a vibratome (Leica, Nussloch, Germany; S100, TPI) was used to cut brains into sections (20 μm), which collected consecutively in PBS for immunohistochemical staining. Free floating sections were blocked with 0.3% H_2_O_2_ in absolute methanol for 30 min, and then, nonspecific sites were blocked with bovine serum albumin (BSA) for 30 min at room temperature. Primary antibody was used to incubate the sections at 4°C overnight. For each primary antibody, 3–5 consecutive sections from each brain were used. Using HistostainTM-SP kits (Zymed, San Francisco, CA. USA), immunoreaction was developed and visualized with diaminobenzidine (brown color). The images were observed using a microscope (Olympus BX60, Tokyo, Japan). We will not use chloral hydrate as an anesthetic for survival surgery and euthanasia for animal welfare in our further animal researches.

### Statistical analysis

The data was expressed as mean±SD or mean±SEM and statistical comparisons were performed using SPSS 12.0 statistical software (SPSS Inc., Chicago, Illinois). Statistical analyses were performed by Student's *t*-test for two-group comparisons. For comparison of multiple groups, 2-way repeated-measures ANOVA or two-way ANOVA with Bonferroni's *post hoc* analysis was used. *P* < 0.05 was accepted as statistically significant in all the experiments.

## Results

### Pp32 levels inversely correlate with learning and memory performance

Since aging is associated with cognitive decline, we utilized a mouse model of aging to explore the role of pp32 in learning and memory. By using 12 month-old mice as the control, we performed MWM-tests to screen out the cognitive-impaired group (Aged-CI) and cognitive-normal group (Aged-CN) from 18-month old aging mice as shown in the methods. Based on their performances in MWM, the cognitive-impaired group (Aged-CI) displayed significantly longer escape latency (>30 s) than 12 m-old mice, and the cognitive-normal group (Aged-CN) exhibited similar escape latency (~20 s) as that of the control [Figure 1A: from day 1 to day 6: Aged-CN vs. Ctrl, group effect: *F*_(1, 16)_ = 1.32, *p* = 0.268; day effect: *F*_(1, 16)_ = 583.423, *p* < 0.001; group × day interaction: *F*_(1, 16)_ = 0.168, *p* = 0.687; Aged-CI vs. Aged-CN, group effect: *F*_(1, 16)_ = 12.269, *p* = 0.003; day effect: *F*_(1, 16)_ = 137.966, *p* < 0.001; group × day interaction: *F*_(1, 16)_ = 2.242, *p* = 0.058 with *p*_4day_ = 0.023, *p*_5day_ = 0.01, *p*_6day_ = 0.04]. Using western blots analysis, we examined pp32 levels in different brain regions including cortex (temporal lobes), hippocampus and cerebellum in Aged-CI, using Aged-CN mice as the control. Pp32 levels were significantly increased in the hippocampus and cortex-brain regions responsible for learning and memory, but not in cerebellum brain region not directly involved in spatial memory in Aged-CI mice, compared to Aged-CN mice (Figures 1B,C: Cr, *p* = 0.002; Hip, *p* = 0.001). The proportion of Aged-CI mice in total aged mice is about 30%. Collectively, these results indicate that pp32 levels inversely correlate with the cognitive performance in aged mice.

### Reducing pp32 level ameliorates cognitive deficits in cognitive-impaired aged mice

**Figure 2:**
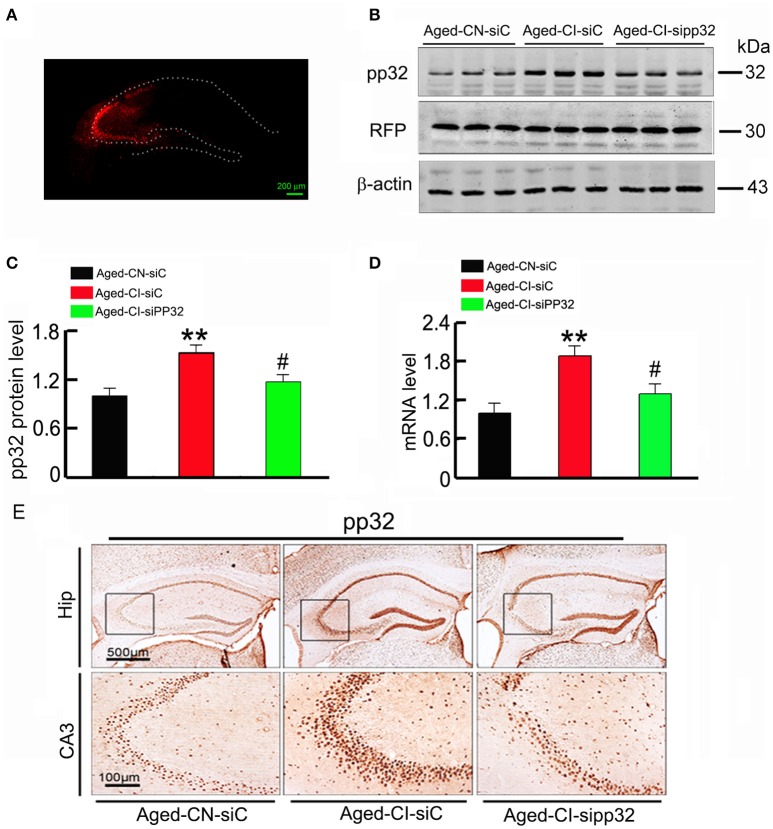
**Sipp32 decreases the protein and mRNA level of pp32 in the hippocampal CA3 region of aged cognitive-impaired mice**. Lentiviruses co-expressing RFP and pp32 shRNA (sipp32) or the scrambled control shRNA (siC) (2 × 10^9^ TU/ml) were stereotaxically injected into the hippocampal CA3 area of Aged-CN or Aged-CI mice (Aged-CN-siC, Aged-CI-siC, and Aged-CI-sipp32). Four weeks later, the mice were sacrificed for the following measurements. **(A)** Representative fluorescent images of LV-transfection in the hippocampal CA3 region of the aged mice. **(B,C)**. Level of pp32 protein and RFP in the hippocampal CA3 region of aged mice was detected by Western blotting and quantitative analysis. **(D)** Level of pp32 mRNA in the hippocampal CA3 region of aged mice was detected by RT-PCR. **(E)** Representative immunohistochemical images of pp32 expression in the hippocampal CA3 region of the aged mice. *N* = 3 for each group. ^**^p < 0.01 vs. Aged-CN-siC; ^#^*p* < 0.05 vs. Aged-CI-siC (mean ± *SD*).

We further determined whether reducing pp32 expression could improve cognitive performance in Aged-CI mice which exhibited behavioral deficits. To this end, lentivirus coexpressing pp32 shRNA (LV-sipp32) and RFP was injected into hippocampal CA3 area of Aged-CI mice to knockdown pp32 expression. The control mice were injected with lentivirus coexpressing scrambled control shRNA (LV-siC) and RFP. The virus infection image was shown in Figure 2A, the RFP level was same in three groups of mice and LV-sipp32 dramatically reduced both the protein[Figures 2B,C: group effect: *F*_(1, 7)_ = 44.121, *p* = 0.001; treatment effect: *F*_(1, 7)_ = 8.464, *p* = 0.027 with p_Aged-*CN-siC vs*. *Aged-CI-siC*_ = 0.001, p_Aged-*CI-sipp*32 *vs*. *Aged-CI-siC*_ = 0.027] and mRNA levels [Figure 2D: group effect: *F*_(1, 7)_ = 28.102, *p* = 0.002; treatment effect: *F*_(1, 7)_ = 11.217, *p* = 0.015 with p_Aged-*CI-siC vs*. *Aged-CN-siC*_ = 0.002, p_Aged-*CI-sipp*32 *vs*. *Aged-CI-siC*_ = 0.015] of pp32 in the hippocampal CA3 of Aged-CI mice. Injection of LV-siC did not significantly affect pp32 expression (Figures 2B–D) Immunohistochemistry assays also showed that the expression of pp32 was much lower in LV-sipp32 injected CA3 subset compared with the LV-siC injection (Figure 2E). So, sipp32 indeed knocked down pp32 expression in Aged-CI mice.

**Figure 3:**
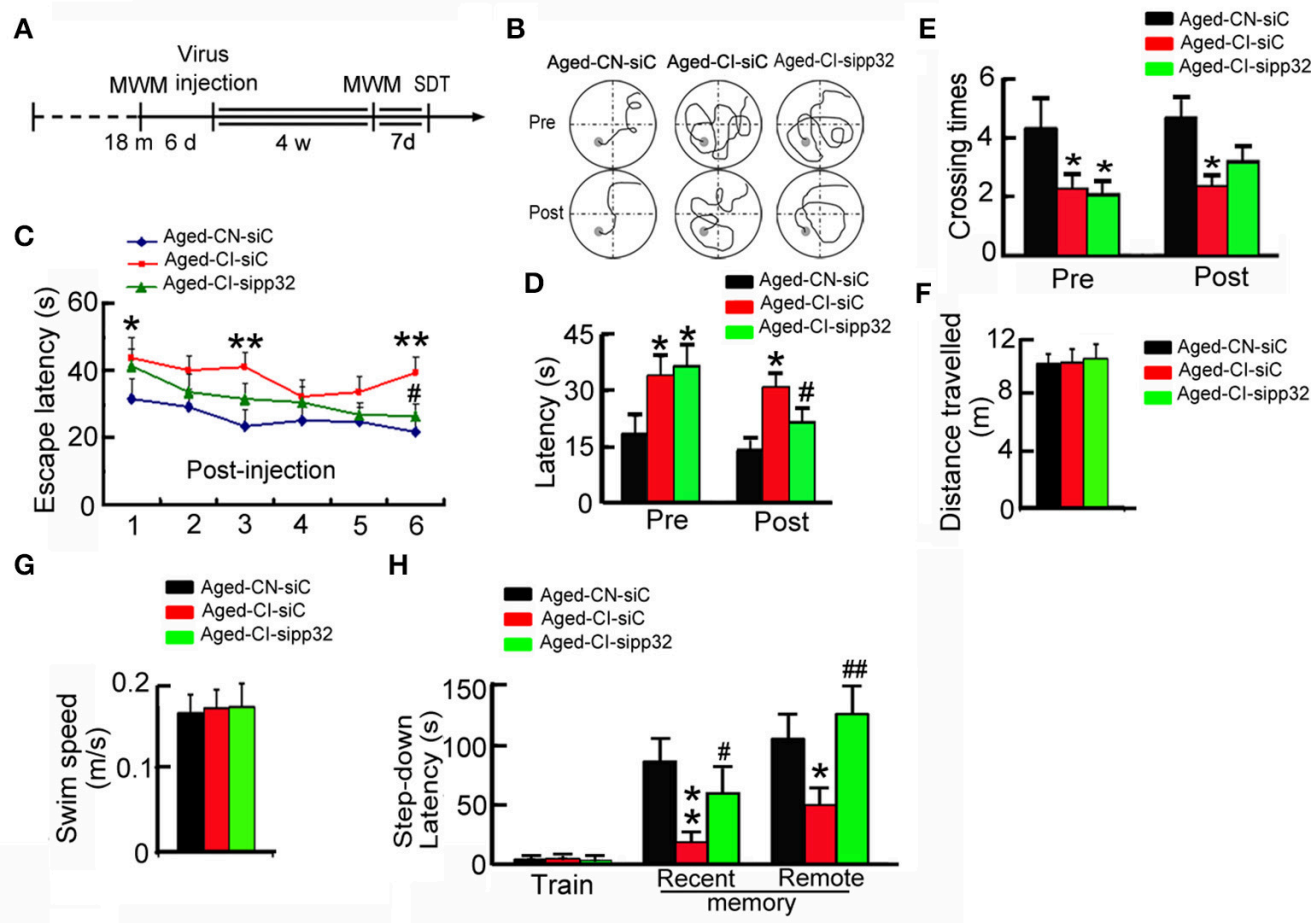
**Silencing pp32 improves learning and memory in Aged-CI mice. (A)** Schematics of experimental procedure for aged mice. **(B)** Representative swim traces of mice in training of MWM task. **(C)** Escape latencies in MWM task of Aged-CN-siC, Aged-CI-siC, and Aged-CI-sipp32 animals. **(D,E)**. Latencies to find the platform **(D)**, and times crossing the place **(E)** where placed the platform during the test. **(F,G)** Distance traveled and swim speed of Aged-CN-siC, Aged-CI-siC and Aged-CI-sipp32 animals in the MWM task. No significant difference was observed among the Aged-CN-siC, Aged-CI-siC, and Aged-CI-sipp32 mice. **(H)** Recent and remote memory were detected by step-down avoidance test. *N* = 11 for each group. ^*^p < 0.05, ^**^p < 0.01 vs. Aged-CN-siC; ^#^*p* < 0.05; ^##^*p* < 0.01 vs. Aged-CI-siC (mean ± SEM).

Next, we determined the influence of knocking down pp32 expression on learning and memory performance by MWM-tests. As before, the MWM was used to prescreen mice into Aged-CI and Aged-CN groups, and then used again 4 weeks after virus injection (Figure 3A).

Before lentivirus injection, the Aged-CI mice showed longer escape latencies to reach the goal than the Aged-CN mice in MWM-tests (Figure 3B). Four weeks later after lentivirus injection, LV-sipp32 not LV-siC injected mice showed dramatically reduced escape latencies during the training [Figure 3C: from day 1 to day 6: Aged-CI-siC vs. Aged-CN-siC, group effect: *F*_(1, 20)_ = 24.157, *p* < 0.001; day effect: *F*_(1, 20)_ = 7.591, *p* = 0.012; group × day interaction: *F*_(1, 20)_ = 0.12, *p* = 0.732 with *p*_1day_ = 0.022, *p*_3day_ = 0.001, *p*_6day_ = 0.002; Aged-CI-sipp32 vs. Aged-CI-siC, group effect: *F*_(1, 20)_ = 8.509, *p* = 0.009; day effect: *F*_(1, 20)_ = 20.277, *p* < 0.001; group × day interaction: *F*_(1, 20)_ = 2.658, *p* = 0.119 with *p*_6day_ = 0.015] and test [Figure 3D: Pre, group effect: *F*_(1, 31)_ = 4.3, *p* = 0.047; treatment effect: *F*_(1, 31)_ = 0.117, *p* = 0.735 with p_Aged-*CI*−*siC vs*. *Aged-CN-siC*_ = 0.047, p_Aged-*CI-sipp*32 *vs*. *Aged-CN-siC*_ = 0.022; Post, group effect: *F*_(1, 31)_ = 14.389, *p* = 0.01; treatment effect: *F*_(1, 31)_ = 4.366, *p* = 0.045 with p_Aged-*CI-siC vs*. *Aged-CN-siC*_ = 0.01, p_Aged-*CI-sipp*32 *vs*. *Aged-CI-siC*_ = 0.045]. Though Aged-CI-sipp32 mice had no difference in times crossing the platform compared to Aged-CN-siC or Aged-CI-siC mice, knockdown pp32 induced aged-CI mice crossed the platform more often during the test [Figure 3E: Pre, group effect: *F*_(1, 31)_ = 4.544, *p* = 0.041; treatment effect: *F*_(1, 31)_ = 0.063, *p* = 0.804 with p_Aged-*CI-siC vs*. *Aged-CN-siC*_ = 0.041, p_Aged-*CI-sipp*32 *vs*. *Aged-CN-siC*_ = 0.024; Post, group effect: *F*_(1, 31)_ = 6.925, *p* = 0.013; treatment effect: *F*_(1, 31)_ = 1.554, *p* = 0.222 with p_Aged-*CI-siC vs*. *Aged-CN-siC*_ = 0.013]. The swimming distance and speed were similar between the different groups (Figures 3F,G). We also analyzed behavior performance using step-down avoidance test. Compared to Aged-CN mice, the Aged-CI mice displayed significantly shorter recent and remote step-down latency, indicating impaired recent and remote memory, which were completely rescued by LV-sipp32 infection [Figure 3H: Recent, group effect: *F*_(1, 25)_ = 17.217, *p* = 0.001; treatment effect: *F*_(1, 25)_ = 4.306, *p* = 0.049 with p_Aged-*CI-siC vs*. *Aged-CN-siC*_ = 0.001, p_Aged-*CI-sipp*32 *vs*. *Aged-CI-siC*_ = 0.049; Remote, group effect: *F*_(1, 25)_ = 4.649, *p* = 0.041; treatment effect: *F*_(1, 25)_ = 14.272, *p* = 0.002 with p_Aged-*CI-siC vs*. *Aged-CN-siC*_ = 0.041, p_Aged-*CI-sipp*32 *vs*. *Aged-CI-siC*_ = 0.002]. These results demonstrate that reducing pp32 levels could rescue cognitive decline in Aged-CI mice. Combined with the data of Figures 1–3, we found that there was a positive correlation of pp32 levels with escape latency at sixth day during training, which suggested that the animals with higher pp32 level in the hippocampus spent longer time to find the platform (Supplementary Figure 1). This result implied that pp32 levels negatively correlated with cognition.

### Sipp32 increases spine density and the expression of synaptic proteins

**Figure 4:**
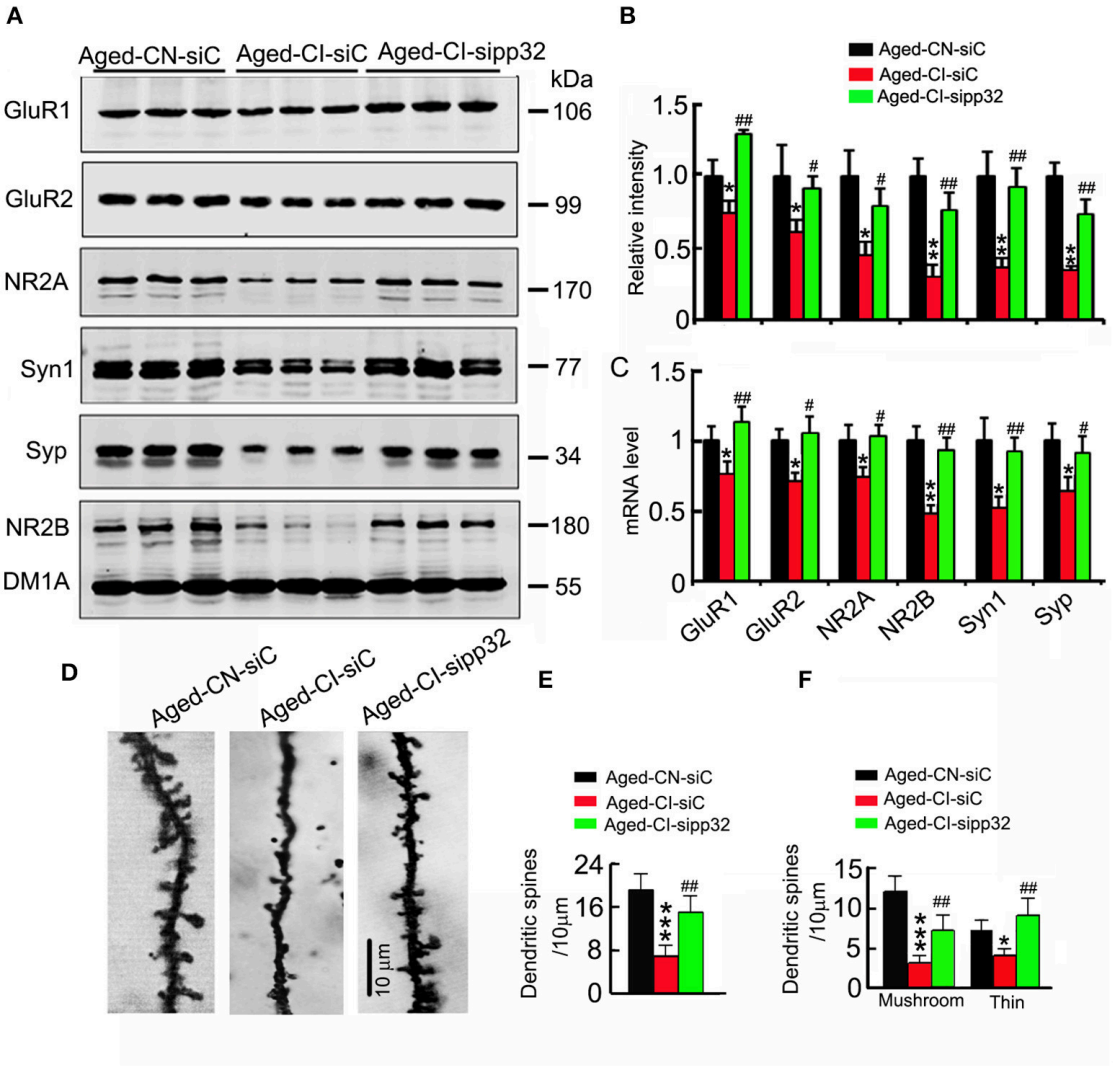
**Sipp32 reverses the impaired spine density and increases the synaptic-related proteins. (A,B)**. The levels of synaptic proteins in the hippocampal CA3 region of Aged-CN-siC, Aged-CI-siC and Aged-CI-sipp32 animals were detected by Western blotting and quantitative analysis (*n* = 3 for each group). **(C)** The mRNA levels of synaptic proteins in the hippocampal CA3 region of Aged-CN-siC, Aged-CI-siC and Aged-CI-sipp32 animals were detected by qRT-PCR (*n* = 3 for each group). **(D,F)** The representative photomicrographs of spines **(D)** and quantitative analysis of spine density **(E)** or density of mushroom- or thin-shaped spines **(F)** in the hippocampal CA3 neurons of Aged-CN-siC, Aged-CI-siC and Aged-CI-sipp32 animals (at least 20 neurons from five to six mice per group). ^*^p < 0.05, ^**^p < 0.01, ^***^p < 0.001 vs. Aged-CN-siC; ^#^*p* < 0.05; ^##^*p* < 0.01 vs. Aged-CI-siC (mean ± *SD*).

Several independent studies have demonstrated that, genes such as N-methyl-D-aspartate receptor type 2A (NR2A) and N-methyl-D-aspartate receptor type 2B (NR2B), AMPA receptor subunits GluR1 and GluR2, as well as synaptophysin (Syp) and synapsin 1 (Syt1) are related to synaptic plasticity (Janz et al., 1999; Fox et al., 2006; Bidoret et al., 2009; Emond et al., 2010; Hutson et al., 2011). We determined the influence of pp32 on the expression of synaptic proteins. The mRNA and protein levels of presynaptic proteins including Syp and Syt1 and postsynaptic proteins including GluR1, GluR2, NR2A, and NR2B were dramatically downregulated in the hippocampal CA3 region of LV-siC injected Aged-CI mice, compared with those in LV-siC injected Aged-CN mice [Figure 4B: group effect: GluR1, *F*_(1, 7)_ = 8.911, *p* = 0.024; GluR2, *F*_(1, 7)_ = 13.21, *p* = 0.011; NR2A, *F*_(1, 7)_ = 12.626, *p* = 0.012; SYN1, *F*_(1, 7)_ = 38.972, *p* = 0.001; SYP, *F*_(1, 7)_ = 9.151, *p* = 0.023; NR2B, *F*_(1, 7)_ = 25.877, *p* = 0.002; treatment effect: GluR1, *F*_(1, 7)_ = 26.862, *p* = 0.002; GluR2, *F*_(1, 7)_ = 6.192, *p* = 0.047; NR2A, *F*_(1, 7)_ = 6.146, *p* = 0.048; SYN1, *F*_(1, 7)_ = 25.004, *p* = 0.002; SYP, *F*_(1, 7)_ = 21.974, *p* = 0.003; NR2B, *F*_(1, 7)_ = 17.97, *p* = 0.005; Aged-CI-siC vs. Aged-CN-siC:p_GluR1_ = 0.024, p_GluR2_ = 0.011, p_NR2A_ = 0.012, p_SYN1_ = 0.001, p_SYP_ = 0.023, p_NR2B_ = 0.002; Aged-CI-sipp32 vs. Aged-CI-siC: p_GluR1_ = 0.002, p_GluR2_ = 0.047, p_NR2A_ = 0.048, p_SYN1_ = 0.002, p_SYP_ = 0.003, p_NR2B_ = 0.005; Figure 4C: group effect: GluR1, *F*_(1, 7)_ = 6.871, *p* = 0.04; GluR2, *F*_(1, 7)_ = 13.073, *p* = 0.011; NR2A, *F*_(1, 7)_ = 6.288, *p* = 0.046; SYN1, *F*_(1, 7)_ = 13.475, *p* = 0.01; SYP, *F*_(1, 7)_ = 35.533, *p* = 0.001; NR2B, *F*_(1, 7)_ = 29.838, *p* = 0.002; treatment effect: GluR1, *F*_(1, 7)_ = 19.186, *p* = 0.005; GluR2, *F*_(1, 7)_ = 30.579, *p* = 0.01; NR2A, *F*_(1, 7)_ = 11.591, *p* = 0.014; SYN1, *F*_(1, 7)_ = 15.957, *p* = 0.007; SYP, *F*_(1, 7)_ = 8.257, *p* = 0.028; NR2B, *F*_(1, 7)_ = 27.486, *p* = 0.002; Aged-CI-siC vs. Aged-CN-siC: p_GluR1_ = 0.04, p_GluR2_ = 0.011, p_NR2A_ = 0.046, p_SYN1_ = 0.01, p_SYP_ = 0.001, p_NR2B_ = 0.002; Aged-CI-sipp32 vs. Aged-CI-siC: p_GluR1_ = 0.005, p_GluR2_ = 0.01, p_NR2A_ = 0.014, p_SYN1_ = 0.007, p_SYP_ = 0.028, p_NR2B_ = 0.002]. Knockdown pp32 with LV-sipp32 significantly elevated the mRNA and protein levels of these synaptic proteins in Aged-CI mice when compared with the control mice (Figures 4A–C).

We also measured spine density of neurons in hippocampal CA3 area. The densities of spines including mushroom and thin-shaped spines in LV-siC injected Aged-CI mice were significantly fewer than those in LV-siC injected Aged-CN mice [Figures 4D–F, Figure 4E: group effect: *F*_(1, 27)_ = 56.593, *p* < 0.001; treatment effect: *F*_(1, 27)_ = 10.211, *p* = 0.004 with p_Aged-*CI-siC vs*. *Aged-CN-siC*_ < 0.001, p_Aged-*CI-sipp*32 *vs*. *Aged-CI-siC*_ = 0.004; Figure 4F: Mushroom, group effect: *F*_(1, 27)_ = 56.876, *p* < 0.001; treatment effect: *F*_(1, 27)_ = 8.622, *p* = 0.007 with p_Aged-*CI-siC vs*. *Aged-CN-siC*_ < 0.001, p_Aged-*CI-sipp*32 *vs*. *Aged-CI-siC*_ = 0.007; Thin, group effect: *F*_(1, 27)_ = 6.381, *p* = 0.018; treatment effect: *F*_(1, 27)_ = 15.003, *p* = 0.001 with p_Aged-*CI-siC vs*. *Aged-CN-siC*_ = 0.018, p_Aged-*CI-sipp*32 *vs*. *Aged-CI-siC*_ = 0.001]. Knockdown of pp32 with LV-sipp32 drastically increased spine densities in Aged-CI mice (Figures 4D–F).

### Pp32 regulates histone acetylation

**Figure 5:**
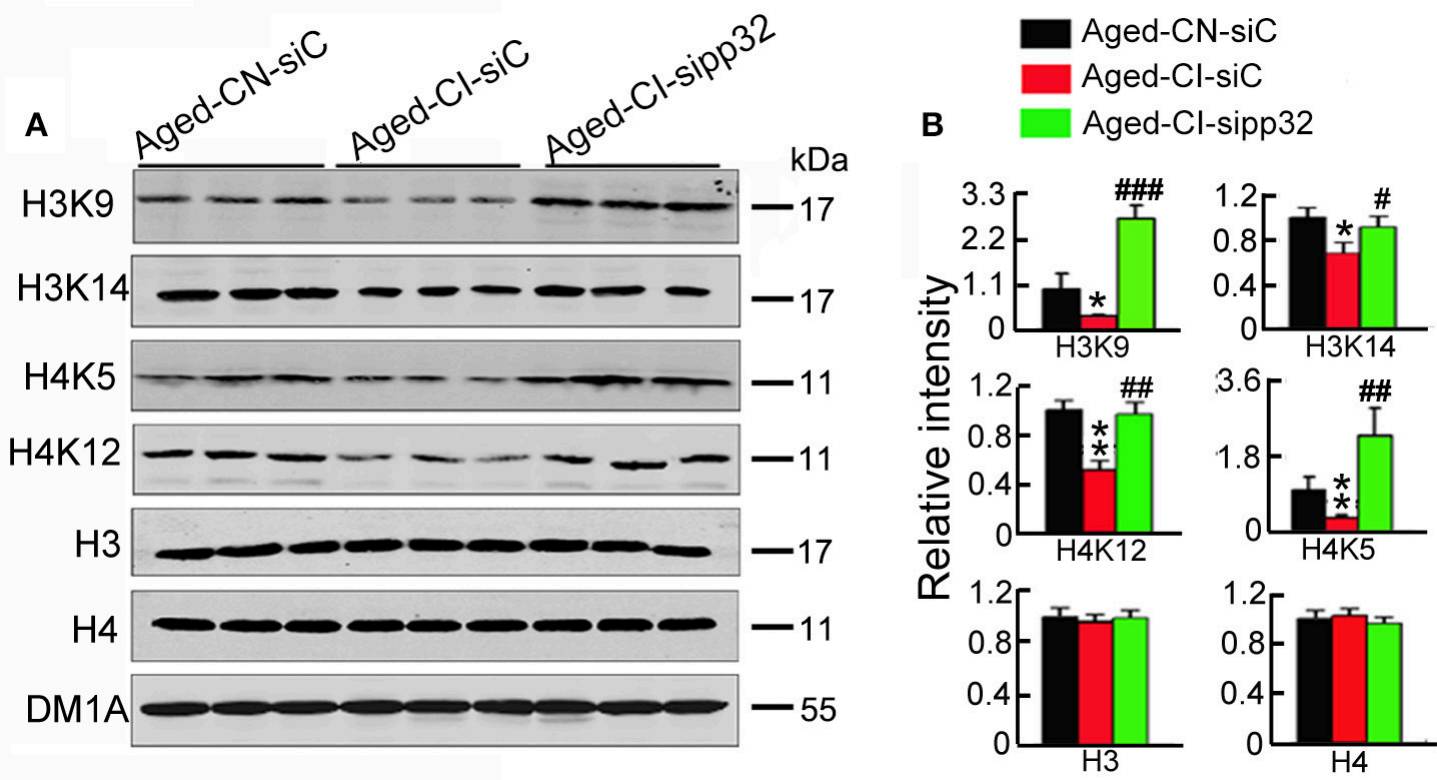
**Sipp32 increases the level of acetylated histone. (A,B)** Western blotting **(A)** and quantitative analysis **(B)** of the level of acetylated histone in the hippocampal CA3 region of Aged-CN-siC, Aged-CI-siC and Aged-CI-sipp32 animals (*n* = 3 for each group). ^*^p < 0.05, ^**^p < 0.01 vs. Aged-CN-siC; ^#^*p* < 0.05; ^##^*p* < 0.01; ^###^*p* < 0.001 vs. Aged-CI-siC (mean ± *SD*).

Pp32 has been implicated in the regulation of histone acetylation (Seo et al., 2002), and histone acetylation could mediate the expression of synaptic proteins involved in synaptic plasticity (Gräff et al., 2012), therefore we assessed whether pp32 could mediate histone acetylation. We particularly focused on histone3 (H3) and histone4 (H4), as they have been linked to the regulation of synaptic protein expression (Gräff et al., 2012). The acetylation of H3 at K9 (H3K9) and K14 (H3K14) or H4 at K5 (H4K5) and K12 (H4K12) was drastically reduced in siC-injected Aged-CI mice, compared with siC-injected Aged-CN mice; knocking down pp32 with sipp32 completely rescued the hypo-acetylation of H3 and H4 in Aged-CI mice, while no significant changes of the levels of total H3 and H4 was observed [Figure 5: group effect: H4K5, *F*_(1, 7)_ = 8.152, *p* = 0.029; H3K14, *F*_(1, 7)_ = 10.061, *p* = 0.019; H3K9, *F*_(1, 7)_ = 16.12, *p* = 0.007; H4K12, *F*_(1, 7)_ = 20.667, *p* = 0.004; H3, *F*_(1, 7)_ = 0.009, *p* = 0.928; H4, *F*_(1, 7)_ = 0.134, *p* = 0.727; treatment effect:H4K5, *F*_(1, 7)_ = 42.437, *p* = 0.001; H3K14, *F*_(1, 7)_ = 6.141, *p* = 0.043; H3K9, *F*_(1, 7)_ = 92.543, *p* < 0.001; H4K12, *F*_(1, 7)_ = 20.101, *p* = 0.004; H3, *F*_((1, 7)_ = 0.272, *p* = 0.621; H4, *F*_(1, 7)_ = 2.326, *p* = 0.178; Aged-CI-siC vs. Aged-CN-siC: p_H4K5_ = 0.029, p_H3K14_ = 0.019, p_H3K9_ = 0.007, p_H4K12_ = 0.004, p_H3_ = 0.928, p_H4_ = 0.727; Aged-CI-sipp32 vs. Aged-CI-siC: p_H4K5_ = 0.001, p_H3K14_ = 0.043, p_H3K9_ < 0.001, p_H4K12_ = 0.004, p_H3_ = 0.621, p_H4_ = 0.178].

### Sipp32 has no significant effects on the phosphorylated level of tau

**Figure 6:**
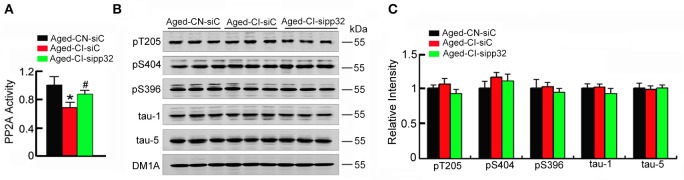
**Sipp32 has no effects on the phosphorylated tau levels. (A)** PP2A activity in the hippocampal CA3 region of Aged-CN-siC, Aged-CI-siC, and Aged-CI-sipp32 animals were measured by using a Serine/Threonine Phosphatase Assay Kit (*n* = 3–5 in triplicates). **(B,C)** Phosphorylated tau protein levels in the hippocampal CA3 region of Aged-CN-siC, Aged-CN-siC, and Aged-CI-sipp32 animals were detected by western blotting and quantitative analysis (*n* = 3 for each group). ^*^p < 0.05, vs. Aged-CN-siC; ^#^*p* < 0.05 vs. Aged-CI-siC (mean ± *SD*).

As pp32 is an endogenous inhibitor of PP2A (Santa-Coloma, 2003), and the reduction of PP2A activity leading to hyperphosphorylation of tau plays an important role in neurodegenerative diseases (Tanimukai et al., 2009), we further detected the phosphorylated level of tau and investigated whether tau phosphorylation contributes to sipp32-ameliorated learning and memory impairment in Aged-CI mice. In Aged-CI mice, PP2A activity was also decreased and knockdown of pp32 increased PP2A activity to ~83% of the control [Figure 6A: group effect: *F*_(1, 7)_ = 13.575, *p* = 0.01; treatment effect: *F*_(1, 7)_ = 6.982, *p* = 0.038 with p_Aged-*CI-siC vs*. *Aged-CN-siC*_ = 0.01, p_Aged-*CI-sipp*32 *vs*. *Aged-CI-siC*_ = 0.038]. Notably, no significant changes of tau phosphorylation were observed in Aged-CI mice, regardless of sipp32 treatment (Figures 6B,C). These data suggest that tau phosphorylation does not play a major role in the altered cognitive function of the Aged-CI mice and after pp32 knockdown.

## Discussion

Aging is a cause of cognitive decline in the elderly and the major risk factor for AD (Yankner, 2000). However, the age-related changes in cognitive function do not correspond well with age-related neuron loss and synaptic change in cortical or temporal structures. Studies have shown that aging affects the expressions of genes related to synaptic plasticity, vesicular transport, and mitochondrial function, such as AMPA receptor subunit GluR1, the NMDA receptor subunit NR2A, VAMP1/synaptobrevin, synapsin II, RAB3A and SNAPs, and so on (Lee et al., 1999; Lu et al., 2004; Xu et al., 2007; Berchtold et al., 2008). In the present study, we divided the aged mice into the cognitive-normal mice and the cognitive-impaired mice by MWM, and we found that synaptic proteins, GluR1, GluR2, NR2A, NR2B, synaptophysin, and synaptotagmin1 in the hippocampus of the cognitive-impaired aging mice were significantly decreased compared to the cognitive-normal mice.

The impairment of histone acetylation has been causally linked to the cognitive decline in aging and a series of neurodegenerative diseases including AD (Peleg et al., 2010; Gräff et al., 2011). HATs and HDACs respectively regulate acetylation and deacetylation of histones. In addition to HATs and HDACs, histone acetylation can be regulated by INHAT, a complex composed of three essential subunits, TAF-Iα, SET/TAF-Iβ, and pp32 (Seo et al., 2001). INHAT regulates histone acetylation by binding to substrate histones thereby preventing their access to HATs (Seo et al., 2001). Pp32, a member of the acidic nuclear phosphoproteins, is a nucleocytoplasmic shuttling protein with a diverse array of functions, including modulation the cytoskeletal compartment by binding to microtubule associated proteins (Ulitzur et al., 1997; Opal et al., 2003). In the nucleus, one of the best characterized functions of pp32 is to inhibit HATs, such as CBP, p300, and PCAF, and conceivably others by binding to the basic histones and preventing HAT access to chromatin (Seo et al., 2002). Pp32 increased in the temporal and entorhinal cortices of the Alzheimer's patients (Tanimukai et al., 2005) and the acetylated levels of histone decreased (Gräff et al., 2012).

The expression of pp32 mRNA is fluctuated in different areas of human brain (Wang et al., 2015). Immunoblot detection revealed that pp32 is expressed throughout the brain, including the hippocampus, temporal cortex, parietal cortex, subcortical nuclei, and brain stem (Kovacech et al., 2007). In the present study, we also found that pp32 is abundant expressed in the hippocampus. The hippocampus plays important roles in the consolidation of information from short-term memory to long-term memory, and in spatial memory. This implies that pp32 may have important functions in neural plasticity for essential acquired ability, such as learning and memory. The hippocampal CA3 region, with its characteristic recurrent collateral connections at the anatomical level, has been proposed as the key structure for the storage of spatial memory or contextual memory (Daumas et al., 2004; Gold and Kesner, 2005). CA3 lesions or experimentally-induced dysfunctions of CA3 induced a severe deficit in spatial memory and fear conditioning (Handelmann and Olton, 1981; Steffenach et al., 2002). Intrahippocampal CA3 information processing is also important for memory-based behavior and can modulate activity in the CA1 (O'Reilly et al., 2014). Here, we chose to decrease pp32 level in CA3.

As pp32 is a component of INHAT, it is suggested that inhibition of INHAT may rescue cognitive function by regulating histone acetylation. In the previous study, histone acetylation and some synaptic protein levels had no change in 3- and 16-month-old naive mice (Peleg et al., 2010). However, we found in the present study that histone acetylation and synaptic protein were changed in aged mice. Two reasons may interpret this difference. First, histone acetylation and synaptic protein levels were detected immediately after the animals were administered step-down avoidance test in the present study; second, the control used here is the ~18-m-old cognitive normal mice, the experiment group mice were also ~18-m-old, but had cognitive impairment; while 3-m-old mice used as the control in the previous study (Peleg et al., 2010).

A recent study found that INHAT domain between amino acids 151 and 180 was a major HAT inhibitory domain of pp32, and was responsible for histone binding, HAT inhibitory activity, and repression of transcription (Seo et al., 2002). By representing pp32 INHAT domain toward the individual histones H2B, H2A, H3, and H4, the relative IC50-values of the synthesized pp32 peptide ranged between 0.6 and 1.5 μM. Though pp32 has a high affinity to bind to and inhibit acetylation of histone H2B and H3, when pp32 is incorporated into the INHAT complex, this H2B preference is lost, and pp32 predominantly binds to and inhibits acetylation of histones H3 and H4 (Seo et al., 2002). Acetylated histones including H3K9, H4K8, and H4K12 were found to bind to the promoter region of the synaptic genes, including glutamate receptor subunits GluR1, GluR2, NR2A, and NR2B, presynaptic protein synaptophysin (Syp) and synaptotagmin 1 (Syt1) (Gräff et al., 2011). Based on these reasons, we detected the acetylated levels of histone H3 and H4, and the protein and mRNA levels of the synaptic gene in the present study. We found that elevation of pp32 decreased the acetylated levels of histone H3 at lysine 9 and 14, and histone H4 at lysine 5 and 12, and the mRNA and protein levels of synaptic genes GluR1, GluR2, NR2A, NR2B, Syp, and Syt1 with decreases of dendritic spine density in the hippocampus of the cognitive-impaired aged mice, resulting in cognitive deficits. However, knockdown pp32 increased the acetylated histone level and the mRNA and protein levels of synaptic genes with increasing dendritic spine density, and resulted in ameliorating cognitive impairments. These results suggest that pp32 plays an important role in learning and memory via epigenetic modifications.

Typically, changes in histone acetylation are associated with changes in transcription, which are necessary for long—but not short-term memory. In the present study, we found that the short-term memory was also affected by pp32 knockdown. This suggests that knockdown pp32 not only affects memory consolidation, but also affects memory acquisition and expression. Previous study reported that depleting pp32 altered 3,187 genes expression including BDNF and GAP43 (Kular et al., 2009), which may explain why sipp32 affects short-term memory here.

One major characteristic pathological change of AD is the intracellular neurofibrillary tangles, which consisted of phosphorylated tau. Pp32 is a multifaceted protein. It is an endogenous inhibitor of PP2A. The up-regulation of pp32 leading to the suppression of PP2A activity has been linked to an increased phosphorylation of tau (Tanimukai et al., 2005). Here we found that phosphorylation level of tau was not altered in cognitive-impaired mice or by pp32 knockdown. These results were consistent with the report showing that mild cognitive decline in aging was in the absence of gross histopathologic changes (Flood et al., 1987a,b; Pakkenberg et al., 2003; Bertoni-Freddari et al., 2007). We speculate that the decreased PP2A activity may be not enough to induce tau phosphorylation. Meanwhile, the effects of the kinases such as GSK-3, also deserves further study.

Previous study reported that knockout pp32 had no significant effect in the cognition in normal mice (Opal et al., 2004). Pp32 null mice were indistinguishable from their wild-type littermates. The mice in 11 week or beyond 12 months performed well on comprehensive neurological tests, including those that test for strength, balance, motor coordination, and learning. Moreover, detailed histological analysis and electrophysiological studies, such as long-term potentiation (LTP) and Paired-pulse facilitation (PPF), did not reveal any major abnormalities (Opal et al., 2004). Therefore, knockdown pp32 in the cognitive-normal mice may not affect cognitive function, which makes pp32 a selective target for AD.

## Conclusions

In conclusion, we found that the level of pp32, one subunit of INHAT involved in epigenetic modification, negatively correlates with cognitive performance in aged mice. Knockdown pp32 increases spine density and ameliorates cognitive deficits by increasing the mRNA and proteins levels of synaptic proteins. Therefore, pp32 (or INHAT) may serve as a potential therapeutic target for learning and memory impairments.

## Author contributions

This study was initiated and designed by JW and GL; JW and GL directed and coordinated the study; QF and GC performed major animal behavior studies, virus injections, Western blotting, and immunohistochemistry; ZW and DS performed brain Golgi staining and dendritic morphology analysis, and collected and analyzed the data; YH and XL performed part of the animal behavior studies and real time PCR; QF and GC analyzed the data; DK. helped to collect and analyze the data; YL and RM performed immunohistochemistry and helped to interpret the results and commented on the manuscript. GL and JW wrote the manuscript. All authors read and approved the final manuscript.

### Conflict of interest statement

The authors declare that the research was conducted in the absence of any commercial or financial relationships that could be construed as a potential conflict of interest.

## References

[B1] Berchtold N. C. Cribbs D. H. Coleman P. D. Rogers J. Head E. Kim R.. (2008). Gene expression changes in the course of normal brain aging are sexually dimorphic. Proc. Natl. Acad. Sci. U.S.A. 105, 15605–15610. 10.1073/pnas.0806883105 18832152 PMC2563070

[B2] Bertoni-Freddari C. Fattoretti P. Giorgetti B. Grossi Y. Balietti M. Casoli T.. (2007). Alterations of synaptic turnover rate in aging may trigger senile plaque formation and neurodegeneration. Ann. N.Y. Acad. Sci. 1096, 128–137. 10.1196/annals.1397.078 17405924

[B3] Bidoret C. Ayon A. Barbour B. Casado M. (2009). Presynaptic NR2A-containing NMDA receptors implement a high-pass filter synaptic plasticity rule. Proc. Natl. Acad. Sci. U.S.A. 106, 14126–14131. 10.1073/pnas.0904284106 19666514 PMC2729031

[B4] Brownell J. E. Allis C. D. (1996). Special HATs for special occasions: linking histone acetylation to chromatin assembly and gene activation. Curr. Opin. Genet. Dev. 6, 176–184. 10.1016/S0959-437X(96)80048-7 8722174

[B5] Chen S. Li B. Grundke-Iqbal I. Iqbal K. (2008). I1PP2A affects tau phosphorylation via association with the catalytic subunit of protein phosphatase 2A. J. Biol. Chem. 283, 10513–10521. 10.1074/jbc.M709852200 18245083 PMC2447634

[B6] Daumas S. Halley H. Lassalle J. M. (2004). Disruption of hippocampal CA3 network: effects on episodic-like memory processing in C57BL/6J mice. Eur. J. Neurosci. 20, 597–600. 10.1111/j.1460-9568.2004.03484.x 15233771

[B7] Duan D. X. Chai G. S. Ni Z. F. Hu Y. Luo Y. Cheng X. S.. (2013). Phosphorylation of tau by death-associated protein kinase 1 antagonizes the kinase-induced cell apoptosis. J. Alzheimers Dis. 37, 795–808. 10.3233/JAD-130377 23948915

[B8] Emond M. R. Montgomery J. M. Huggins M. L. Hanson J. E. Mao L. Huganir R. L.. (2010). AMPA receptor subunits define properties of state-dependent synaptic plasticity. J. Physiol. 588(Pt 11), 1929–1946. 10.1113/jphysiol.2010.187229 20351044 PMC2901981

[B9] Flood D. G. Buell S. J. Horwitz G. J. Coleman P. D. (1987a). Dendritic extent in human dentategyrus granule cells in normal aging and senile dementia. Brain Res. 402, 205–216. 10.1016/0006-8993(87)90027-8 3828793

[B10] Flood D. G. Guarnaccia M. Coleman P. D. (1987b). Dendritic extent in human CA2–3 hippocampalpyramidal neurons in normal aging and senile dementia. Brain Res. 409, 88–96. 10.1016/0006-8993(87)90744-X 3580872

[B11] Fox C. J. Russell K. I. Wang Y. T. Christie B. R. (2006). Contribution of NR2A and NR2B NMDA subunits to bidirectional synaptic plasticity in the hippocampus *in vivo*. Hippocampus 16, 907–915. 10.1002/hipo.20230 17024679

[B12] Gold A. E. Kesner R. P. (2005). The role of the CA3 subregion of the dorsal hippocampus in spatial pattern completion in the rat. Hippocampus 15, 808–814. 10.1002/hipo.20103 16010664

[B13] Gräff J. Kim D. Dobbin M. M. Tsai L. H. (2011). Epigenetic regulation of gene expression in physiological and pathological brain processes. Physiol. Rev. 91, 603–649. 10.1152/physrev.00012.2010 21527733

[B14] Gräff J. Rei D. Guan J. S. Wang W. Y. Seo J. Hennig K. M.. (2012). An epigenetic blockade of cognitive functions in the neurodegenerating brain. Nature 483, 222–226. 10.1038/nature10849 22388814 PMC3498952

[B15] Handelmann G. E. Olton D. S. (1981). Spatial memory following damage to hippocampal CA3 pyramidal cells with kainic acid: impairment and recovery with preoperative training. Brain Res. 217, 41–58. 10.1016/0006-8993(81)90183-9 7260619

[B16] Hutson P. H. Finger E. N. Magliaro B. C. Smith S. M. Converso A. Sanderson P. E. (2011). The selective phosphodiesterase 9(PDE9) inhibitor PF-04447943(6-[(3S, 4S)-4-methyl-1-(pyrimidin-2-ylmethyl) pyrrolidin-3-yl]-1-(tetrahydro-2H-pyran-4-yl)-1, 5-dihydro-4H-pyrazolo [3, 4-d] pyrimidin-4-one) enhances synapticplasticity and cognitive function in rodents. Neuropharmacology 61, 665–676. 10.1016/j.neuropharm.2011.05.009 21619887

[B17] Janz R. Südhof T. C. Hammer R. E. Unni V. Siegelbaum S. A. Bolshakov V. Y. (1999). Essential roles in synaptic plasticity for synaptogyrin I and synaptophysin I. Neuron 24, 687–700. 10.1016/S0896-6273(00)81122-8 10595519

[B18] Jenuwein T. Allis C. D. (2001). Translating the histone code. Science 293, 1074–1080. 10.1126/science.1063127 11498575

[B19] Kosik K. S. Rapp P. R. Raz N. Small S. A. Sweatt J. D. Tsai L. H. (2012). Mechanisms of age-related cognitive change and targets for intervention: epigenetics. J. Gerontol. A Biol. Sci. Med. Sci. 67, 741–746. 10.1093/gerona/gls110 22522509 PMC4009730

[B20] Kovacech B. Kontsekova E. Zilka N. Novak P. Skrabana R. Filipcik P. (2007). A novel monoclonal antibody DC63 reveals that inhibitor 1 of proteinphosphatase 2A is preferentially nuclearlylocalised in human brain. FEBS Lett. 581, 617–622. 10.1016/j.febslet.2007.01.015 17266954

[B21] Kular R. K. Cvetanovic M. Siferd S. Kini A. R. Opal P. (2009). Neuronal differentiation is regulated by leucine-rich acidic nuclear protein (LANP), a member of the inhibitor of histone acetyltransferase complex. J. Biol. Chem. 284, 7783–7792. 10.1074/jbc.M806150200 19136565 PMC2658072

[B22] Lee C. K. Klopp R. G. Weindruch R. Prolla T. A. (1999). Gene expression profile of aging and its retardation by caloric restriction. Science 285, 1390–1393. 10.1126/science.285.5432.1390 10464095

[B23] Li M. Makkinje A. Damuni Z. (1996). Molecular identification of I1PP2A, a novel potent heat-stable inhibitor protein of protein phosphatase 2A. Biochemistry 35, 6998–7002. 10.1021/bi960581y 8679524

[B24] Liu G. P. Wei W. Zhou X. Shi H. R. Liu X. H. Chai G. S.. (2013). Silencing PP2A inhibitor by lenti-shRNA interference ameliorates neuropathologies and memory deficits in tg2576 mice. Mol. Ther. 21, 2247–2257. 10.1038/mt.2013.189 23922015 PMC3863796

[B25] Lu T. Pan Y. Kao S. Y. Li C. Kohane I. Chan J.. (2004). Gene regulation and DNA damage in the ageing human brain. Nature 429, 883–891. 10.1038/nature02661 15190254

[B26] Magariños A. M. Li C. J. Gal Toth J. Bath K. G. Jing D. Lee F. S.. (2011). Effect of brain-derived neurotrophic factor haploinsufficiency on stress-induced remodeling of hippocampal neurons. Hippocampus 21, 253–264. 10.1002/hipo.20744 20095008 PMC2888762

[B27] Marintcheva B. Marintchev A. Wagner G. Richardson C. C. (2008). Acidic C-terminal tail of the ssDNA-binding protein of bacteriophage T7 and ssDNA compete for the same binding surface. Proc. Natl. Acad. Sci. U.S.A. 105, 1855–1860. 10.1073/pnas.0711919105 18238893 PMC2538852

[B28] Morris R. G. Garrud P. Rawlins J. N. O'Keefe J. (1982). Place navigation impaired in rats with hippocampal lesions. Nature 297, 681–683. 10.1038/297681a0 7088155

[B29] Opal P. Garcia J. J. McCall A. E. Xu B. Weeber E. J. Sweatt J. D.. (2004). Generation and characterization of LANP/pp32 null mice. Mol. Cell. Biol. 24, 3140–3149. 10.1128/MCB.24.8.3140-3149.2004 15060138 PMC381670

[B30] Opal P. Garcia J. J. Propst F. Matilla A. Orr H. T. Zoghbi H. Y. (2003). Mapmodulin/leucine-rich acidic nuclear protein binds the light chain of microtubule-associated protein 1B and modulates neuritogenesis. J. Biol. Chem. 278, 34691–34699. 10.1074/jbc.M302785200 12807913

[B31] O'Reilly K. C. Alarcon J. M. Ferbinteanu J. (2014). Relative contributions of CA3 and medial entorhinal cortex to memory in rats. Front. Behav. Neurosci. 8:292. 10.3389/fnbeh.2014.00292 25221487 PMC4148030

[B32] Pakkenberg B. Pelvig D. Marner L. Bundgaard M. J. Gundersen H. J. Nyengaard J. R.. (2003). Aging and the human neocortex. Exp. Gerontol. 38, 95–99. 10.1016/S0531-5565(02)00151-1 12543266

[B33] Peleg S. Sananbenesi F. Zovoilis A. Burkhardt S. Bahari-Javan S. Agis-Balboa R. C.. (2010). Altered histone acetylation is associated with age-dependent memory impairment in mice. Science 328, 753–756. 10.1126/science.1186088 20448184

[B34] Santa-Coloma T. A. (2003). Anp32e(Cpd1) and related protein phosphatase 2 inhibitors. Cerebellum 2, 310–320. 10.1080/14734220310017212 14964690

[B35] Seo S. B. Macfarlan T. McNamara P. Hong R. Mukai Y. Heo S.. (2002). Regulation of histone acetylation and transcription by nuclear protein PP32, a subunit of the INHAT complex. J. Biol. Chem. 277, 14005–14010. 10.1074/jbc.M112455200 11830591

[B36] Seo S. B. McNamara P. Heo S. Turner A. Lane W. S. Chakravarti D. (2001). Regulation of histone acetylation and transcription by INHAT, a human cellular complex containing the set oncoprotein. Cell 104, 119–130. 10.1016/S0092-8674(01)00196-9 11163245

[B37] Spires-Jones T. L. Kay K. Matsouka R. Rozkalne A. Betensky R. A. Hyman B. T. (2011). Calcineurin inhibition with systemic FK506 treatment increases dendritic branching and dendritic spine density in healthy adult mouse brain. Neurosci. Lett. 487, 260–263. 10.1016/j.neulet.2010.10.033 20970476 PMC3010509

[B38] Steffenach H. A. Sloviter R. S. Moser E. I. Moser M. B. (2002). Impaired retention of spatial memory after transection of longitudinally oriented axons of hippocampal CA3 pyramidal cells. Proc. Natl. Acad. Sci. U.S.A. 99, 3194–3198. 10.1073/pnas.042700999 11867718 PMC122495

[B39] Tanimukai H. Grundke-Iqbal I. Iqbal K. (2005). Up-regulation of inhibitors of protein phosphatase-2A in Alzheimer's disease. Am. J. Pathol. 166, 1761–1771. 10.1016/S0002-9440(10)62486-8 15920161 PMC1602412

[B40] Tanimukai H. Kudo T. Tanaka T. Grundke-Iqbal I. Iqbal K. Takeda M. (2009). Novel therapeutic strategies for neurodegenerative disease. Psychogeriatrics 9, 103–109. 10.1111/j.1479-8301.2009.00289.x 19604333 PMC3139458

[B41] Ulitzur N. Rancaño C. Pfeffer S. R. (1997). Biochemical characterization of mapmodulin, a protein that binds microtubule-associated proteins. J. Biol. Chem. 272, 30577–30582. 10.1074/jbc.272.48.30577 9374554

[B42] Wang S. Wang Y. Lu Q. Liu X. Wang F. Ma X.. (2015). The expression and distributions of ANP32A in the developing brain. Biomed. Res. Int. 2015:207347. 10.1155/2015/207347 25866766 PMC4383345

[B43] Woolley C. S. McEwen B. S. (1992). Estradiol mediates fluctuation in hippocampal synapse density during the estrous cycle in the adult rat. J. Neurosci. 12, 2549–2554. 1613547 10.1523/JNEUROSCI.12-07-02549.1992 PMC6575846

[B44] Xu X. Zhan M. Duan W. Prabhu V. Brenneman R. Wood W.. (2007). Gene expression atlas of the mouse central nervous system: impact and interactions of age, energy intake and gender. Genome Biol. 8:R234. 10.1186/gb-2007-8-11-r234 17988385 PMC2258177

[B45] Yankner B. A. (2000). A century of cognitive decline. Nature 404:125. 10.1038/35004673 10724145

