# Supplementary material for: Knockdown of pp32 Increases Histone Acetylation and Ameliorates Cognitive Deficits
Source: Front Aging Neurosci. 2017 Apr 20;9:104. doi: 10.3389/fnagi.2017.00104 (PMC5397422; doi:10.3389/fnagi.2017.00104)
Supplement: Supplementary file 1 [file Image1.pdf]

Supplementary Fig. 1

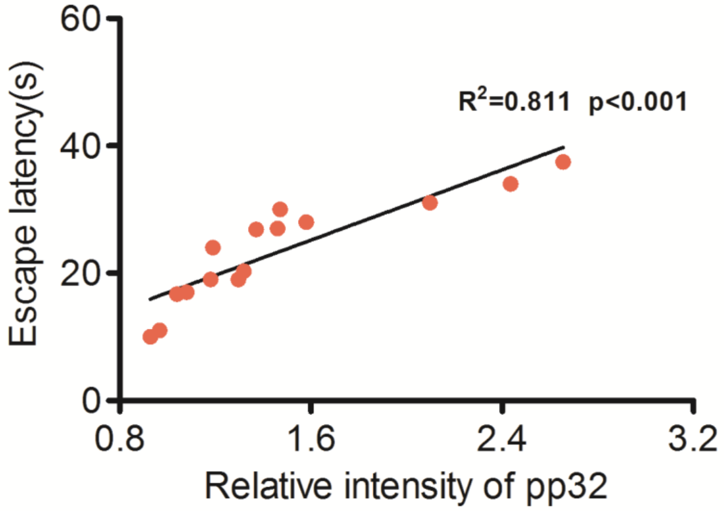

Fig. S1 Pp32 levels negatively correlated with cognitive performance.

Combined the data of Fig. 1-3, there is a highly positive correlation between escape latency at 6th day during training with the corresponding protein level of pp32 in the hippocampus of mice (n=15), which suggested that the animals with higher pp32 level in the hippocampus spent longer time to find the platform.
